# Incomplete tumour control following DNA vaccination against rat gliomas expressing a model antigen

**DOI:** 10.1007/s00701-012-1526-7

**Published:** 2012-11-08

**Authors:** Christian Ginzkey, Sven Eicker, Matthias Marget, Jörg Krause, Stefan Brecht, Manfred Westphal, Heinz-Hermann Hugo, Maximilian Mehdorn, Jörg Steinmann, Wolfgang Hamel

**Affiliations:** 1 Department of Neurosurgery, University of Schleswig-Holstein, Campus Kiel, Kiel, Germany; 2 Institute of Immunology, University of Schleswig-Holstein, Campus Kiel, Kiel, Germany; 3 Institute of Pharmacology, University of Schleswig-Holstein, Campus Kiel, Kiel, Germany; 4 Department of Oto-Rhino-Laryngology, Julius-Maximilian-University, Würzburg, Germany; 5 Department of Neurosurgery, Heinrich-Heine-University, Düsseldorf, Germany; 6 Department of Neurosurgery, University Hospital Hamburg-Eppendorf, Hamburg, Germany; 7 Klinik für Neurochirurgie, Universitätsklinikum Hamburg-Eppendorf, Martinistr. 52, 20246 Hamburg, Germany

**Keywords:** DNA vaccination, Immunotherapy, Rat glioma, lacZ

## Abstract

**Background:**

Vaccination against tumour-associated antigens is one approach to elicit anti-tumour responses. We investigated the effect of polynucleotide (DNA) vaccination using a model antigen (E. coli lacZ) in a syngeneic gliosarcoma model (9L).

**Methods:**

Fisher 344 rats were vaccinated thrice by intramuscular injection of a lacZ-encoding or a control plasmid in weekly intervals. One week after the last vaccination, lacZ-expressing 9L cells were implanted into the striatum.

**Results:**

After 3 weeks, in lacZ-vaccinated animals the tumours were significantly smaller than in control-vaccinated animals. In cytotoxic T cell assays lysis rates of >50 % could only be observed in a few of the lacZ-vaccinated animals. This response was directed against lacZ-expressing and parental 9L cells but not against syngeneic MADB 106 adenocarcinoma cells. In Elispot assays interferon-γ production was observed upon stimulation with 9LlacZ and 9L wild-type but not MADB 106 cells. This response was higher for lacZ-immunized animals. All animals revealed dense infiltrates with CD8+ lymphocytes and, to a lesser extent, with NK cells. CD25-staining indicated cells possibly associated with the maintenance of peripheral tolerance to self-antigens. All tumours were densely infiltrated by microglia consisting mostly of ramified cells. Only focal accumulation of macrophage-like cells expressing ED1, a marker for phagocytic activity, was observed.

**Conclusion:**

Prophylactic DNA vaccination resulted in effective but incomplete suppression of brain tumour formation. Mechanisms other than cytotoxic T cell responses as measured in the generally used in vitro assays appear to play a role in tumour suppression.

## Introduction

Malignant gliomas cannot be cured despite technical developments aiding surgical resection techniques, optimised radiotherapy, and novel (local and systemic) chemotherapy. However, we have obtained broad knowledge regarding molecular alterations involved in glioma formation and tumour maintenance. This has fostered the search for novel adjuvant therapies, including gene therapy and immunotherapy, which have been pursued in several preclinical and clinical studies. However, a relevant survival benefit has not been achieved to date [2, 4].

Malignant gliomas are highly infiltrative tumours contributing to their inevitable recurrence. Specific activation of the immune system has always been regarded as a worthwhile attempt in order to eliminate residual tumour cells by immuno surveillance. Several approaches have been utilised, including transfer of adoptive T cells, peptide immunization, and vaccination with dendritic cells pulsed with tumour-derived proteins or nucleic acids [9, 16].

Polynucleotide (DNA) vaccination represents another straightforward approach with possible advantages. In contrast to immunisation with peptides, DNA vaccination appears to result in stronger cytotoxic, Th1-mediated responses that are regarded as crucial for effective anti-tumour effects [3, 18]. Furthermore, the vaccine, i.e., the expression plasmids, can be readily produced in large quantities, and it is possible to immunise with the whole cDNA, obviating the need to characterise and synthesise specific epitopes. To evaluate this vaccination concept in a brain tumour model, we used a model antigen (E. coli lacZ) for repeated intramuscular vaccination prior to intracerebral implantation of E. coli lacZ-expressing glioma cells in a syngeneic rat model.

## Materials and methods

### Cell lines

The 9L rat gliosarcoma cells were obtained from the Brain Tumour Research Center, University of California, San Francisco, CA, USA. MADB 106 rat adenocarcinoma cells were a kind gift from Dr. Thomas von Hörsten, Medizinische Hochschule Hannover, Germany. Both cell lines are syngeneic to Fisher 344 rats. 9LlacZ cells had been transfected with the LNPOZ vector (kindly provided by Dr. A.D. Miller, Seattle, WA) containing the E. coli lacZ gene and the neomycin resistance gene. The latter served for selection of stable lacZ-expressing cells by selection with G418 after transfection with Effectene^TM^ (Qiagen, Hilden, Germany). Cells were cultured in DMEM supplemented with 2 mM L-glutamine, 1,000 mg/l D-glucose, and 2 mM sodium pyruvate (GIBCO BRL Life Technologies, Karlsruhe, Germany; 9L and 9LlacZ cell lines) or RPMI (GIBCO BRL Life Technologies, Karlsruhe, Germany; MADB 106 cell lines) containing both 10 % heat-inactivated foetal calf serum and 1 % penicillin/streptomycin (Sigma-Aldrich, St. Louis, MO, USA) at 37 °C in a humid atmosphere with 5 % CO_2_.

### Plasmids

DNA vaccination against the lacZ antigen was performed with a lacZ-containing expression vector (pcDNA3.1/His B/lacZ; Invitrogen, Karlsruhe, Germany). Control animals were injected with an empty expression vector (pcDNA3.1/His B; Invitrogen, Karlsruhe, Germany). To prevent vaccination against the neomycin resistance gene, this gene had been deleted from both plasmids, and vector integrity was confirmed by sequence analysis. Large-scale preparation of plasmid DNA was performed with the EndoFree GigaPrep® (Qiagen, Hilden, Germany). DNA was solved in 0.9 % sterile saline and stored in aliquots at –20 °C.

### Vaccination protocol and tumour cell implantation

Male Fisher 344 rats (250 to 275 g) were purchased from Charles River (Sulzfeld, Germany). Animals were housed according to German Animal Protection Regulations, and permission had been obtained from the local authorities. Weekly vaccinations into both anterior tibial muscles were performed thrice with 100 μg DNA per leg in a volume of 50 μl normal saline. One week after the last vaccination, animals were anaesthetised with 4 % chloral hydrate (1 ml per 100 g) and the heads were mounted into a stereotactic frame (TSE Systems, Bad Homburg, Germany). A burr hole was placed 3 mm lateral and 1 mm anterior to the bregma, and 2 × 10^4^ 9L/lacZ cells suspended in 5 μl DMEM without supplements were slowly injected into the right striatum with a 10-μl Hamilton syringe. The needle was carefully retracted and the burr hole was sealed with bone wax. After 3 weeks animals were killed and spleens were removed under sterile conditions and kept in ice-cold RPMI containing 10 % FCS until lymphocyte preparation. Blood was collected from the right atrium, and brains were removed and transferred into anti-freeze medium (Reichert-Jung, Nussloch, Germany), shock-frozen in liquid nitrogen, and stored at –20 °C.

### Histology and immunohistochemistry

The tumour volume was calculated from serial hemalaun-eosin-stained sections (200 μm distance) using the following formula: V = 4/3 x π × 0.125 (length × height × width). Coronal sections of 10 μm were cut with a cryostat 2800 Frigocut (Reichert-Jung, Nussloch, Germany), and we identified sections where the tumour appeared first and where it disappeared. For immunohistochemical and X-Gal staining, brain slices were mounted on coated slides (Marienfeld GmbH, Lauda-Königshofen, Germany), air-dried, and stored at –20 °C in aluminium foil.

The 9L cells expressing the lacZ gene convert X-Gal to 5,5′-dibromo-4,4′-dichloro-indigo, staining the cytoplasm of these cells blue. Brain slices were fixed with 10 % formaldehyde for 10 min and washed twice with PBS. Staining with X-Gal was performed in a moist chamber at 37 °C overnight. Slides were washed in PBS twice for 5 min and covered with Aquatex (Merck, Darmstadt, Germany).

Immunohistochemical staining was performed according to Barclay et al. [1]. Slices were fixed in acetone at – 20 °C for 10 min and rinsed in tap water for 1 min. To eliminate endogenous peroxidase, slices were transferred into PBS/methanol (1:1) containing 0.3 % H_2_O_2_ and washed thrice with PBS. Immunohistochemistry was performed with the following mouse anti-rat monoclonal antibodies (obtained from Serotec, Oxford, UK; all antibodies diluted 1:400): W3/25 (anti-rat CD4), MRC OX-8 (anti-rat CD8), MRC OX-39 (anti-rat CD25), R73 (anti-rat αβ T-cell receptor), 10/78 (anti-rat NKR-P1), and ED1 (anti-rat CD68). In addition, the following rabbit anti-rat polyclonal antibodies were used: anti-perforin (Torrey Pine Biolabs, San Diego, CA; dilution 1:400) and Iba1 directed against an ionised calcium-binding adaptor molecule-1 expressed in microglia and macrophages (kind gift from Y. Imai, Department of Neurochemistry, National Institute of Neuroscience, Tokyo; dilution 1:1,500). Incubation with the primary antibodies in PBS with 1 % bovine serum albumin (Sigma-Aldrich, Munich, Germany) was performed at 4 °C overnight. Slices were washed three times with PBS and incubated with the secondary biotinylated rabbit anti-mouse or swine anti-rabbit antibody (Dako, Hamburg, Germany; 1:400), which was added in PBS with 10 % heat-inactivated rat serum at 37 °C for 1 h. Slices were washed and incubated with a streptavidine complex (Dako, Hamburg, Germany; 1:300) in PBS at 37 °C for 45 min, and 3,3′-diaminobenzidine (DAB; Dako, Hamburg, Germany) served as the chromogene substrate. After nuclear staining with hemalaun (Merck, Darmstadt, Germany), slices were covered with Aquatex (Merck, Darmstadt, Germany).

### Cytotoxic T lymphocyte assay

Spleens were removed under sterile conditions and transferred to a petri dish (Becton Dickinson Labware, Franklin Lakes, NJ, USA) to generate a cell suspension that was passed through a 70-μm-pore filter (Becton Dickinson). The cell suspension was layered onto Lympholyte M (Cedar Lane, Ontario, Canada), and mononuclear cells were isolated by density-gradient centrifugation with 1,000 g for 20 min. After washing thrice, cells were transferred to a petri dish, and cells were allowed to adhere to the bottom at 37 °C for 2 h. In contrast to monocytes/macrophages and B cells that adhere to plastic, T cells can be collected by aspiration off the culture media after gentle shaking. Cells were washed twice with RPMI, and their viability was determined by trypan blue staining. Usually 1-2 × 10^8^ mononuclear cells per spleen were obtained.

Generation of effector cells for the chromium release assay was performed as described previously [17, 20]. In brief, 9L/lacZ cells serving as stimulator cells were seeded in 6-well plates and irradiated with a lethal dose of 40 Gy. T cell-enriched mononuclear cells (5 × 10^6^) were added at a ratio of 1:10, which proved optimal in preliminary experiments, and co-cultures were incubated for 7 days. On days 3 and 5, fresh RPMI (10 % FCS, penicillin G/streptomycin) containing 30 U/ml human recombinant Il-2 (Sigma-Aldrich, Munich, Germany) was added.

On day 7, target cells (9L/lacZ, 9Lwt, or MADB106; 5 × 10^5^ cells) were labelled with 200 μCi Na^51^CrO_4_ (Amersham-Buchler, Braunschweig, Germany) in 1 ml RPMI containing 10 % FCS in a shaking water bath at 37 °C for 1 h. After washing thrice to eliminate non-incorporated Na^51^CrO_4_, cells were counted and viability rates of >90 % were assured by trypan blue staining. Target cells (TC) were adjusted to 30,000 cells/ml, distributed on 96-well round-bottom plates (Corning Incorporated, Corning, NY, USA), and let to adhere for 2–3 h before effector cells were added. Effector cells (EC) were collected, counted, and added to labelled TC in fresh RPMI in different ratios (10:1, 20:1, 40:1, 80:1) in triplicate. Contact between target and effector cells was achieved by centrifugation of plates for 3 min. Plates were incubated at 37 °C for 4 h. The radioactive supernatant containing released ^51^Cr was soaked off with cotton wool (Scatron Titertek harvesting system; Scatron, Suffolk, UK) and transferred to a Gamma counter (Canberra-Packard, Frankfurt, Germany). Spontaneous release of ^51^Cr was determined from TC without exposure to EC (equivalent to 0 % specific release). Maximum (100 %) release of ^51^Cr was determined following TC lysis with 10 % Triton-X detergent. Specific lysis was calculated as follows:

$$ \mathrm{Specific}\ \mathrm{lysis}\ \left( \% \right)=100\times \left[ {{{{\left( {\mathrm{experimental}\ \mathrm{cpm}\text{-} \mathrm{spontaneous}\ \mathrm{cpm}} \right)}} \left/ {{\left( {\mathrm{maximum}\ \mathrm{cpm}\text{-} \mathrm{spontaneous}\ \mathrm{cpm}} \right)}} \right.}} \right] $$

### IFN-γ synthesis (Elispot assay)

IFN-γ synthesis by stimulated T-cells was determined with a commercially available Elispot kit (Diaclone, Besançon, France) following the protocol provided by the manufacturer with minor modifications according to Heiser et al. [10]. In brief, PVDF 96-well plates were incubated with an anti-rat IFN-γ antibody (capture antibody) at 4 °C overnight. The next day, freshly isolated spleen cells (5 × 10^5^ responder cells per well) were washed and resuspended in RPMI containing 10 % FCS and co-incubated in the INF-γ antibody-precoated 96-well plates with 9L/lacZ, 9Lwt, and MADB106 (10^5^ stimulator cells per well). After incubation at 37 °C for 20 h, the cells were removed from the plate, and a biotinylated anti-rat IFN-γ antibody (detection antibody) was added and detected with streptavidine-conjugated alkaline phosphatase converting the substrate BCIP/NBT to a blue dye. Dots were counted using the Bioreader system (BIO-SYS GmbH, Karben, Germany).

## Results

**Fig. 1:**
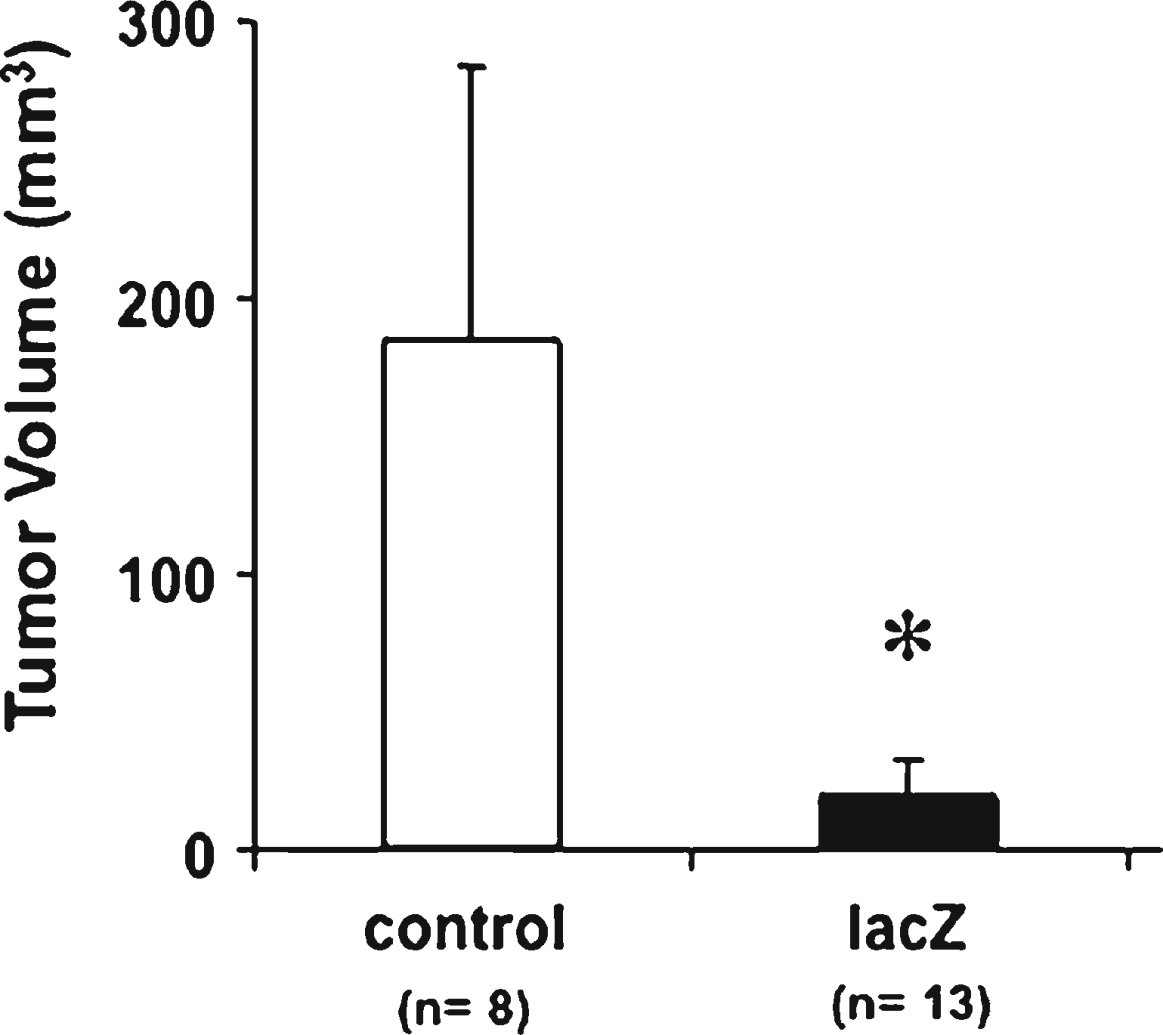
Rat glioma tumour volumes 3 weeks after intracerebral implantation of 9L lacZ cells following DNA vaccination. Animals had been vaccinated thrice with an empty expression vector (‘control’) or a lacZ-encoding vecor (‘lacZ’) in weekly intervals followed by intracerebral tumour cell implantation. The number of treated animals is indicated. Tumour volumes (mean and standard deviation) were determined from serial coronal sections. *p< 0.001 (Student’s t test). In the lacZ group, one animal died because of apnea during anaesthesia for tumour cell implantation. In the control group, two animals died because of excessive tumour growth before the experiment had been terminated. Massive tumour growth was verified by histology, although post-mortem tissue artefacts prevented accurate measurements

**Fig. 2:**
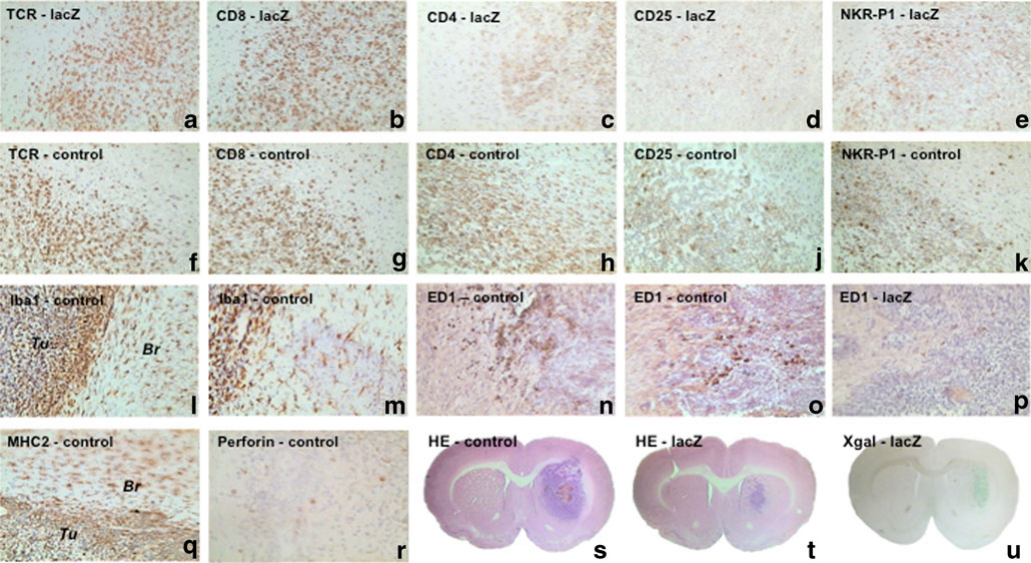
Immunohistochemical characterisation of T-cell infiltrates and microglial cells detected in tumours from control-vaccinated and lacZ-vaccinated animals. Tumours of control-vaccinated and lacZ-vaccinated animals were heavily infiltrated with TCR+ cells (**a** and **f**) that were predominantly CD8+ lymphocytes (**b** and **g**). The same region also contained moderate NK cell infiltrates (**e** and **k**). Staining for CD4 revealed dense infiltrates with cells representing CD4+ lymphocytes and microglial cells, which could also be detected in the peritumoral brain parenchyma (**c** and **h**). Microglial cells stained MHC 2 positive (**q**), and staining for a microglial-specific marker (Iba1) revealed the abundance of microglial cells both within the tumour and in the peritumoral brain parenchyma (**l** and **m**). ED1 staining, a marker indicating phagocytic activity, mostly revealed focal expression (**n**–**p**), in particular in necrotic regions (**n**). Perforin serving as a marker for cytotoxic activity of NK and CD8+ cells revealed only a few positive cells within the tumour (**r**). The cells staining positive for CD25 probably represent regulatory CD4+ cells involved in the maintenance of peripheral tolerance (**d** and **j**). Representative coronal brain sections were stained with haematoxylin and eosin, indicating large tumours in control animals (**s**) and markedly smaller tumours in the lacZ-vaccinated animals (**t** and **u**). The smaller tumours in the lacZ-vaccinated animals stained with X-Gal, indicating that selection against the lacZ antigen had not occurred (**u**)

Intramuscular polynucleotide vaccination performed thrice with a lacZ expression plasmid prior to implantation of lacZ expressing tumour cells was associated with a strong anti-tumour response. Whereas control vaccinated animals revealed large tumours (183.7 mm^3^; SD 99.2), the residual tumours in lacZ-vaccinated animals were significantly smaller (18.9 mm^3^; SD 13.3; *p*<0.05) (Fig. 1). Representative tumours are shown in Fig. 2. Necrotic areas and neovascularisation were observed, but almost no infiltrating tumour growth (Fig. 2 s–u). Vaccination with saline (no plasmid) resulted in the formation of tumours similar in size to control vaccinated animals (data not shown). In the lacZ group, one animal died because of apnea during anaesthesia for tumour cell implantation. In the control group, two animals died from excessive tumour growth before the experiment was terminated. In both animals, large tumours were found at autopsy, although exact tumour volumes could not be determined because of post-mortem artefacts.

Immunohistochemical staining revealed strong lymphocytic and microglial cell infiltrates in tumours of all animals (Fig. 2). Regardless of the type of prior vaccination (control vs. lacZ vector), the pattern of infiltration did not differ. Dense T-cell infiltrates (TCR staining) were found within tumours of both lacZ- (Fig. 2a) and control-vaccinated (Fig. 2f) animals, which was pronounced at the tumour margins and extended into peritumoral normal brain. Characterisation of T-cell infiltrates showed predominantly CD8+ T cells (Fig. 2 b and g). Staining for CD4 revealed both lymphocytes as well as dense infiltrates with microglial cells (Fig. 2c and h). All tumours also revealed NK cells that were less abundant than T cells (Fig. 2e and k). Perforin granula suggesting cytolytic activity of NK cells and T cells could only be detected in single cells (Fig. 2r). All tumours contained CD25+ cells, which probably represent regulatory CD4+ (or CD8+) T cells involved in peripheral tolerance to self-antigens (Fig. 2d and j).

Two types of microglial cells could be distinguished following staining with Iba1, MHC 2, and ED1 (Fig. 2l-q). First and foremost, all tumours were densely infiltrated with microglial cells revealing a ramified phenotype. Such cells could be detected by staining against the MHC2 or Iba1 antigen (Fig. 2 l,m,q). Besides this, small clusters of macrophage-like cells were detected (Fig. 2 n–p). These cells stained positive for ED1, which is detected on lysosomal membranes of cells of the mononuclear phagocytosis system (Fig. 2 n–p). Thus, ED1 appears to represent a microglial subpopulation with phagocytic activity or macrophages. In general, the intensity of ED1 staining was most prominent around necrotic areas (Fig. 2n). Nonetheless, islets of ED1-positive cells were also found in non-necrotic areas (Fig. 2 o and p).

X-Gal staining was performed to assess β-galactosidase expression in residual tumours (Fig. 2u). Positive staining suggests that residual tumours in lacZ-vaccinated animals were not due to selection against the lacZ gene (Fig. 2u).

**Fig. 3:**
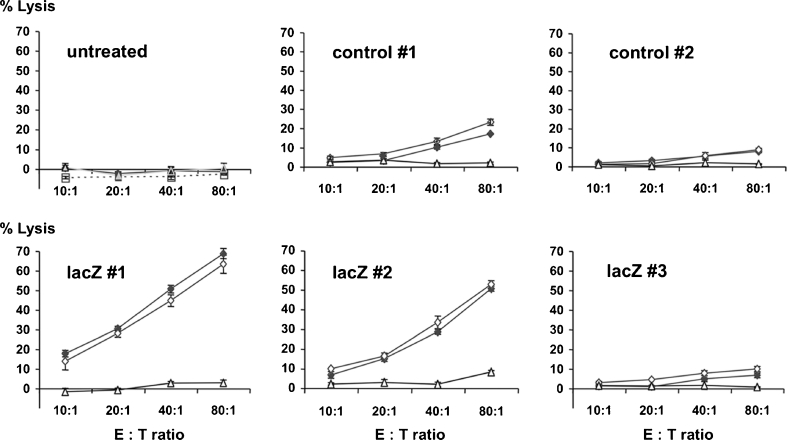
Cytotoxic T cell assays with lymphocytes prepared from spleens of untreated, control-vaccinated, or lacZ-vaccinated animals. Splenocytes were restimulated for 6 days with 9LlacZ cells and added to ^51^Cr-labelled target cells (9LlacZ, filled diamonds; 9L wild-type, open diamonds; MADB, open triangles). The percentage of target cell (TC) lysis in relation to different effector-target cell ratios (E:T-Ratio) is shown for representative animals of each group. Whereas high levels of ^51^Cr-release (>50 %) could only be observed in lacZ-vaccinated animals (lacZ #1 and lacZ #2), specific but weaker lysis (<25 %) of 9LlacZ and parental 9L cells was also detected in control-vaccinated animals (control #1). DNA vaccination against lacZ did not result in a lacZ-restricted response. CTL activity directed against 9LlacZ cells was always paralleled by a similar response against the parental 9L cell line. However, cytotoxic activity was restricted to the 9L cell line and not observed with another syngeneic cell line (MADB)

To evaluate cytotoxic T cell activity directed against the implanted tumours, CTL assays were performed with lymphocytes generated from in vitro restimulated spleen cell preparations. As determined by flow cytometry, >70 % of the restimulated cells were CD3-positive lymphocytes (approximately 33 % CD8+ and 52 % CD4+ cells). Restimulated cells contained less than 1 % cells that stained positive for NKR-P1, which was used to identify natural killer cells (data not shown). Strong cytotoxic responses with lysis rates of >50 % were only observed in few animals, all of which had been vaccinated against the lacZ antigen (Fig. 3). This cytotoxicity was specific for both 9LlacZ cells and the parental 9L cell line (Fig. 3). No cell lysis was observed when another syngeneic cell line, MADB 106 rat adenocarcinoma, was used as target cells (Fig. 3). Although some lacZ-vaccinated animals revealed strong cytotoxic activity, this did not correlate with tumour size or lymphocytic infiltrates as well as CD25 or perforin staining. Cell lysis was T cell receptor (TCR)-dependent since addition of a monoclonal antibody against the rat TCR (R73; 1:200) 1 h prior to target cell exposure suppressed release of labelled chromium by >50 % (not shown). Less inhibition was observed with monoclonal antibodies directed against MHC 1 (30 % inhibition), CD8 (20 %), or CD4 (20 %).

Specific responses directed against 9LlacZ and parental 9L cells (but not MADB 106) were also observed in several animals of the control group (Fig. 3). These animals had not been vaccinated against the lacZ antigen but exposed to 9LlacZ cells (intracerebral tumours). In control animals, however, the lysis rates remained below 25 % (Fig. 3). Lymphocytes derived from naive animals neither vaccinated nor exposed to tumour cells did not elicit cytolytic activity against any of the three target cell lines (Fig. 3).

**Fig. 4:**
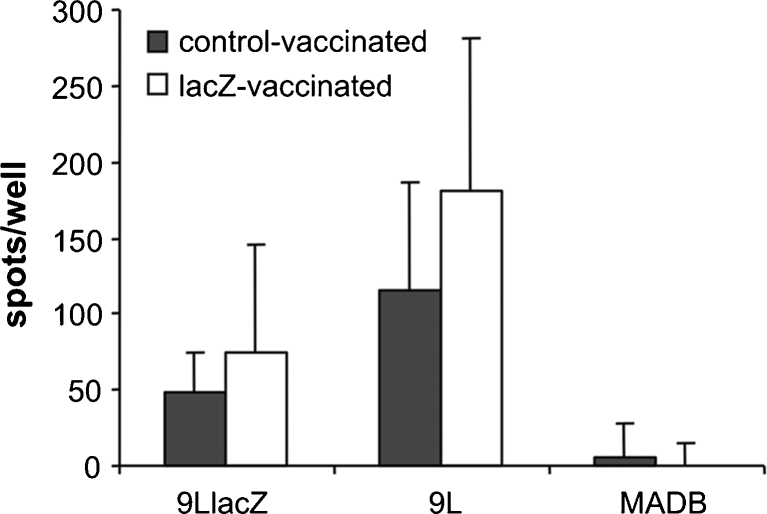
Quantification of IFN-γ synthesis by the Elispot assay performed with splenocytes isolated from control-vaccinated (*filled bars*) or lacZ-vaccinated (*open bars*) animals. IFN-γ synthesis was observed in lymphocytes derived from both control-vaccinated and lacZ-vaccinated animals following exposure to 9LlacZ cells or the parental 9L cell line for 20 h. No such response was observed with a syngeneic rat adenocarcinoma cell line (MADB). Although IFN-γ production was higher in lacZ-vaccinated animals, this did not reach statistical significance (*p*>0.5; ANOVA). The amount of IFN-γ detected was higher following exposure to 9L cells compared to 9LlacZ cells. *Bars* indicate standard deviations

To further investigate anti-tumour immune responses, IFN-γ synthesised by splenic lymphocytes exposed to 9LlacZ, 9L wild-type or MADB 106 cells was quantified by Elispot analysis. IFN-γ was produced by lymphocytes from lacZ-vaccinated as well as control-vaccinated animals when stimulated with 9LlacZ or the parental 9L cell line, but not following exposure to syngeneic MADB 106 cells (Fig. 4). The amount of IFN-γ synthesized was higher upon stimulation with 9L than 9LlacZ cells, which was independent of the vaccination status (Fig. 4). Although prior lacZ vaccination resulted in higher IFN-γ synthesis with both 9LlacZ or wild-type 9L stimulator cells, this was not statistically significant (*p* > 0.05; ANOVA). The level of IFN-γ production by lymphocytes of individual animals and the size of their tumours did not correlate.

## Discussion

This study demonstrates that intramuscular DNA vaccination against a model antigen (lacZ) suppresses the formation of intracerebral tumours in a syngeneic rat model. Whereas in the control vaccinated animals large tumours were detected (including two animals which had died from excessive tumour growth), in the lacZ-vaccinated animals only small tumours had formed. We did not quantify the efficacy of vector uptake at the vaccination site. Although this appears unlikely, we cannot rule out that more effective uptake of the lacZ expression plasmid in conjunction with unspecific (lacZ-independent) immune stimulation was resposible for the decreased tumour size in the lacZ-vaccinated animals.

We chose a point of time 3 weeks after intracerebral tumour cell inoculation to assess tumour growth since this was sufficient for the formation of large tumours in the control-vaccinated group. Although at this point significantly smaller tumours were found in the vaccinated animals, tumour formation had not been prevented completely. We cannot rule out that the small tumours detected in the vaccinated animals on the day of sacrifice represent tumour remnants during an ongoing process of tumour rejection. However, it appears more likely that after completed preventive vaccination tumour rejection had occurred directly after tumour cell inoculation (i.e. solid tumour formation had been prevented altogether). The fact that solid tumours were observed at all argues in favour of an insufficient immune response merely delaying or retarding tumour growth. Others have reported long-term survival in a murine brain tumour model following DNA vaccination [12]. The effect of vaccination and reduced tumour growth on suvival time was not investigated in our model.

To investigate immune mechanisms possibly involved in the anti-tumour effects observed immunohistochemical staining and immunological in vitro assays (CTL and Elispot assay) were employed. DNA vaccination was required for lysis rates of >50 % in CTL assays, which, however, were only observed in a few animals. Similarly, IFN-γ synthesis as quantified by Elispot assays was higher in lacZ-vaccinated animals, although this did not reach statistical significance. Thus, DNA vaccination resulted in the priming of specific cytotoxic responses as expected from previous reports [13, 15, 21]. Despite vaccination against the lacZ antigen, this response was not restricted to lacZ-expressing cells, but included the parental cell line. We did not restimulate the lymphocytes with the parental 9L cell line. Thus, it is unresolved to what extent restimulation with the antigen proper (lacZ) was required for effective target cell lysis in those animals revealing a strong CTL response. The fact, however, that parental 9L (target) cells were lysed with the same efficacy as 9LlacZ (target) cells suggests that the lacZ antigen proper was not crucial for effective restimulation. A possible mechanism is antigen spreading within the 9L (but not to the syngeneic MADB 106) context.

Notably, intracerebral tumour cell implantation following vaccination with a control plasmid also elicited 9L tumour-specific cytotoxic responses in vitro, although this response was weaker. Thus, lacZ vaccination may augment an intrinsic immune response present in the 9L tumour model occurring independently from prior immunisation. This response was specific for 9L tumours and not observed with another syngeneic adenocarcinoma cell line (MADB 106).

Although in a few of the animals pronounced responses in both in vitro assays had been observed, this response did not correlate with the anti-tumour effect of prior vaccination. Thus, cytotoxic T cell responses not detectable by CTL and Elispot assays or even unidentified effector mechanisms may play a role in tumour suppression.

This is supported by the fact that the immunohistochemical staining pattern of the different treatment groups was indistinguishable. Tumour size did not correlate with the degree of lymphocytic infiltration or activation. Rather heterogeneous staining patterns were observed within individual tumours, e.g. more pronounced infiltrations around necrotic areas. This is consistent with the ambiguous role proposed for the well-recognised lymphocytic and microglial infiltrations in malignant gliomas [8, 14, 19]. In fact, only single cells stained positive for perforin serving as a marker for cytolytic activity in situ. We detected immunoreactivity for CD25 (interleukin-2 receptor alpha chain), known to be expressed by activated T and B cells, macrophages, and CD4+ and CD8+ regulatory T cells. As recognised in recent years, in gliomas and other tumours CD25+ cells often represent regulatory T cells (FoxP3+), and these cells have been demonstrated to play an important role in the maintenance of peripheral tolerance [5, 6, 11]. The exact role of different CD25+ cell populations in tumours, however, is not fully understood. Since staining for CD25 did not differ between lacZ- and control-vaccinated animals, we did not attempt to distinguish different subpopulations of CD25+ cells.

All tumours were densely infiltrated with microglial cells. Only few microglial cells stained ED-1 positive, indicating phagocytic activity. Such cells were predominantly found in necrotic regions. The majority of microglial cells revealed a more ramified morphology. There is accumulating evidence that these cells are in the service of the tumour [8, 14, 19]. Microglial cells appear to promote tumour growth directly (e.g. by producing growth factors) as well as indirectly by the secretion of immunosuppressive cytokines (e.g. TGF-β, IL-10) and the expression of molecules inducing apoptosis in lymphocytes (e.g. Fas ligand). The latter factors contribute to the local and systemic immunosuppression and peripheral tolerance observed in gliomas.

Because of this we attempted to augment the anti-tumour response by intratumoral application of oligonucleotides containing unmethylated CpG motifs because of their known macrophage/microglia-activating properties. Such oligonucleotides exerted no adjuvant effect; on the contrary, they resulted in an increase in tumour size in the 9L model. This was observed both following vaccination against the lacZ gene (data not shown) and in non-vaccinated animals challenged with naive 9L cells [7]. Furthermore, we evaluated the adjuvant effects of flt-3 ligand and IL-12 expression plasmids that had been added to the lacZ plasmid used for vaccination (data not shown). Coadministration of both plasmids did not increase the anti-tumour response elicited by lacZ vaccination only, but, in contrast, there was a trend to the formation of larger tumours.

In this study we used a therapeutic regimen easily applicable to patients. Despite a robust anti-tumour effect, there are several reasons for being cautious with regard to possible clinical efficacy in glioma patients. We used an idealised setting with prophylactic vaccination in a non-infiltrative rodent model that is known to respond to different immunotherapeutic approaches. Nevertheless, we could not prevent tumour formation and the failure of all adjuvants tested thus far reflects how unpredictable and counter-productive their effects can be. This does not even touch on the issue of whether vaccination against a single antigen is sufficient, although, in our model we did not observe tumour escape due to selection against the model antigen.
